# K_Ca_3.1-Dependent Hyperpolarization Enhances Intracellular Ca^2+^ Signaling Induced by fMLF in Differentiated U937 Cells

**DOI:** 10.1371/journal.pone.0139243

**Published:** 2015-09-29

**Authors:** Antonello Penna, Andrés Stutzin

**Affiliations:** Instituto de Ciencias Biomédicas, Facultad de Medicina, Universidad de Chile, Independencia 838–0453, Santiago, Chile; University at Buffalo, UNITED STATES

## Abstract

Formylated peptides are chemotactic agents generated by pathogens. The most relevant peptide is fMLF (formyl-Met-Leu-Phe) which participates in several immune functions, such as chemotaxis, phagocytosis, cytokine release and generation of reactive oxygen species. In macrophages fMLF-dependent responses are dependent on both, an increase in intracellular calcium concentration and on a hyperpolarization of the membrane potential. However, the molecular entity underlying this hyperpolarization remains unknown and it is not clear whether changes in membrane potential are linked to the increase in intracellular Ca^2+^. In this study, differentiated U937 cells, as a macrophage-like cell model, was used to characterize the fMLF response using electrophysiological and Ca^2+^ imaging techniques. We demonstrate by means of pharmacological and molecular biology tools that fMLF induces a Ca^2+^-dependent hyperpolarization via activation of the K^+^ channel K_Ca_3.1 and thus, enhancing fMLF-induced intracellular Ca^2+^ increase through an amplification of the driving force for Ca^2+^ entry. Consequently, enhanced Ca^2+^ influx would in turn lengthen the hyperpolarization, operating as a positive feedback mechanism for fMLF-induced Ca^2+^ signaling.

## Introduction

Formylated peptides are by-products produced by pathogens capable to induce immune cell chemotaxis during an immune response [1, 2]. The most relevant formylated peptide is fMLF (formyl-Met-Leu-Phe) produced mainly by *Escherichia coli* [3]. fMLF stimulates formylated peptide receptor 1 (FPR1) with high affinity (EC_50_ = 0.1–1 nM) and formylated peptide receptor 2 (FPR2) with low affinity (EC_50_ = 1 mM) [1, 2] triggering downstream activation of G_αi_ and G_βγ_ subunits [1, 2, 4] and thus, promoting several cellular functions aimed to eliminate pathogens, such as chemotaxis, phagocytosis, cytokine release and generation of reactive oxygen species [2, 5, 6].

fMLF-dependent effects in macrophages and neutrophils are mediated by an increase in intracellular calcium concentration ([Ca^2+^]_i_) [7–9] and by changes in the membrane potential (V_m_) [10, 11]. fMLF increases [Ca^2+^]_i_ by activating IP_3_ receptors causing Ca^2+^ release from intracellular reservoirs [7, 12]. On the other hand, it has been reported that fMLF hyperpolarizes V_m_ (-15 to -60 mV) in macrophages [10]. This hyperpolarization has been also observed in neutrophils and was found to be dependent on a Ca^2+^-activated K^+^ channel [13, 14]. However, the molecular entity underlying this hyperpolarization remains unknown. Moreover, from a mechanistic point of view it is not clear whether changes in V_m_ increases further [Ca^2+^]_i_ by modulating the driving force for Ca^2+^ entry.

The intermediate-conductance Ca^2+^-activated K^+^ channel K_Ca_3.1 [15–17] is expressed in immune cells [18–20]. This channel is activated by an increase in [Ca^2+^]_i_ leading to an hyperpolarization of the V_m_ [21]. In macrophages, a K_Ca_3.1-dependent hyperpolarization triggered by external ATP and mediated by an increase in [Ca^2+^]_i_ has been previously described [20]. Similarly, in microglia K_Ca_3.1 activation occurs by P2Y2 receptor stimulation triggering intracellular Ca^2+^ signaling [22]. Thus, K_Ca_3.1 appears as a molecular candidate responsible for the hyperpolarization induced by fMLF. In this study, we used differentiated U937 cells as a macrophage cell model, to characterize the fMLF response. We determined that K_Ca_3.1 is indeed responsible for the fMLF-induced hyperpolarization and modulation of the driving force for Ca^2+^ entry.

## Material and Methods

### Cell culture

The U937 cell line from American Type Cell Culture (ATCC, Catalogue CRL-1593.2) was kindly provided to us by C. Allers, Universidad del Desarrollo, Santiago, Chile. Cells were grown as a cellular suspension at 37°C and humidified 5% CO_2_ atmosphere in RPMI 1640 medium (Gibco, Grand Island, NY, USA) supplemented with 10% heat-inactivated fetal bovine serum (FBS, Gibco) and 100 units/mL penicillin-streptomycin (HyClone, Waltham, MA, USA). Cells were maintained at a concentration of 0.5x10^6^ to 5x10^6^ cells/mL and three times per week the medium was changed. Differentiation of U937 cells was induced with dibutyryl cAMP [23, 24]. Cells (0.3–0.5x10^6^ cells/mL) were incubated with 1 mM of dibutyryl cAMP for 48 h resulting in mature adherent monocytes expressing FPR [24]. After 48 h cells were washed with PBS and were maintained in RPMI 1640 medium until experiments were performed (on the same day).

### K_Ca_3.1 knock down

K_Ca_3.1 knockdown was achieved by infecting U937 cells with a set of three different lentiviral particles carrying GFP-tagged human KCNN4 shRNA (catalogue number VSH6286-00EG3783, GE Healthcare Dharmacon, Inc., Chicago, IL, USA). Cells were infected as indicated by the manufacturer. Selection was achieved with puromycin electrophysiological experiments were performed in green cells one week after infection.

### Electrophysiological measurements

Nystatin perforated patch-clamp experiments were performed after 48 h of differentiation. Cells were plated on 12-mm coverslips and directly mounted on a chamber (RC-25, Warner Instruments Corp., Hamden CT, USA) fitted on the stage of an inverted microscope (Diaphot, Nikon Inc., Melville, NY,USA). Internal pipette solution contained (in mM): NaCl 5, KCl 140, MgCl_2_ 1, HEPES 10 and pH 7.2 adjusted with TRIS, nystatin 165 μg/mL was freshly prepared and added to the pipette solution. For the experiment depicted in S2 Fig the pipette solution was modified replacing 140 mM KCl with 140 mM CsCl. The bath solution contained (in mM): NaCl 140, KCl 5, MgCl_2_ 1, CaCl_2_ 2, HEPES 10, D-glucose 10 and pH 7.4 adjusted with NaOH. The low Na^+^ bath solution used contained (in mM): NaCl 5, NMDGCl 100, KCl 5, MgCl_2_ 1, CaCl_2_ 2, HEPES 10, sorbitol 50, D-glucose 10 and pH 7.4 adjusted with NaOH. The 105 mM K^+^ solution contained (in mM): NaCl 5, KCl 105, MgCl_2_ 1, CaCl_2_ 2, HEPES 10, sorbitol 50, D-glucose 10 and pH 7.4 adjusted with NaOH. The bath solution without Ca^2+^ was prepared adding 5 mM EGTA in absence of CaCl_2_. The 65 mM K^+^ solution was prepared adding 65 mM KCl and 80 mM NaCl. Bath solutions were changed by a gravity-fed perfusion system and the solution level in the chamber was kept constant by a peristaltic pump. Voltage- and current-clamp experiments were recorded with EPC-7 amplifier (List-Medical, Darmstadt, Germany). Data were digitized at 10 kHz and low-pass filtered at 1 kHz. pClamp 8.0 software (Molecular Device Corp., Sunnyvale, CA, USA) was used for data acquisition and analysis. Patch electrodes (4 MΩ resistance) were pulled from borosilicate glass (Warner Instruments) using a BB-CH puller (Mecanex SA, Geneva, Switzerland). All experiments were performed at room temperature.

### Calcium measurements

Intracellular Ca^2+^ concentration changes were measured using the Ca^2+^ indicators Fluo-3 AM, Fluo-4 AM and Indo-1 AM (Invitrogen, Waltham, MA, USA), as indicated in the figures. U937 cells after 48 h of differentiation were incubated with 2 μM of the given indicators dissolved in RPMI 1640 without FBS for 30 min in the dark at 37^°^C. Cells were then kept for 30 min in the presence of RPMI 1640 with 10% FBS in the dark. U937 cells were washed 2 times with PBS and suspended or bathed with the same solutions used and described for the electrophysiological measurements. Cells were exposed to the different drugs, [K^+^], EGTA or control conditions for approximately 5 min before starting the fluorescence measurements. fMLF was perfused throughout the experiments as shown in each figure. Fluorescence was measured using three different systems depending on the type of experiment and Ca^2+^ indicator used. Sinergy 2 multi-mode microplate reader (BioTek, Winooski, VT, USA) was used to measured fluorescence changes in 50–100x10^3^ U937 cells for each condition and acquisition was made using Gene5 Data Analysis software (BioTek). Disk Spinning Confocal Unit (Olympus Corp., Tokyo, Japan) was used to quantify fluorescence changes in the cytoplasm of U937 cells and Cell v2.8 software (Olympus Corp.) was used for acquisition. Two fluorometers were used to measured fluorescence changes in U937 cells incubated with Indo-1 AM and acquisition was performed with AxoScope 8.2 software (Molecular Devices). For Fluo-3 and Fluo-4 the cells were excited at 485 nm and emission was detected at 520 nm. Fluorescence changes were expressed as (Ft-F0)/F0, where Ft was the fluorescence at any time and F0 was the basal fluorescence during 2 min before stimulus. For Indo-1 cells were excited at 340 nm and the acquisition was made at 405 nm and 485 nm. Fluorescence changes were expressed as the ratio 405/485 and the results were expressed as R1/R0, where R1 was the ratio at any time and R0 was the basal ratio during 2 min before stimulus.

### Reagents

All reagents were of analytical grade and were purchased from Sigma (St. Louis, MO, USA) and Merck (Darmstadt, Germany). fMLF and dibutyryl cAMP were purchased from Sigma (St. Louis, MO, USA) and dissolved in DMSO.

### Statistics

Data are represented as mean ± SEM. Unpaired Student’s *t* test was used to compare between two groups. For comparing three or more groups, one-way analysis of variance (ANOVA) followed by Bonferroni's *post-hoc* test was used. Statistical significance was set at *p* < 0.05.

## Results

### fMLF increases [Ca^2+^]_i_ and hyperpolarizes U937 cells

**Fig 1:**
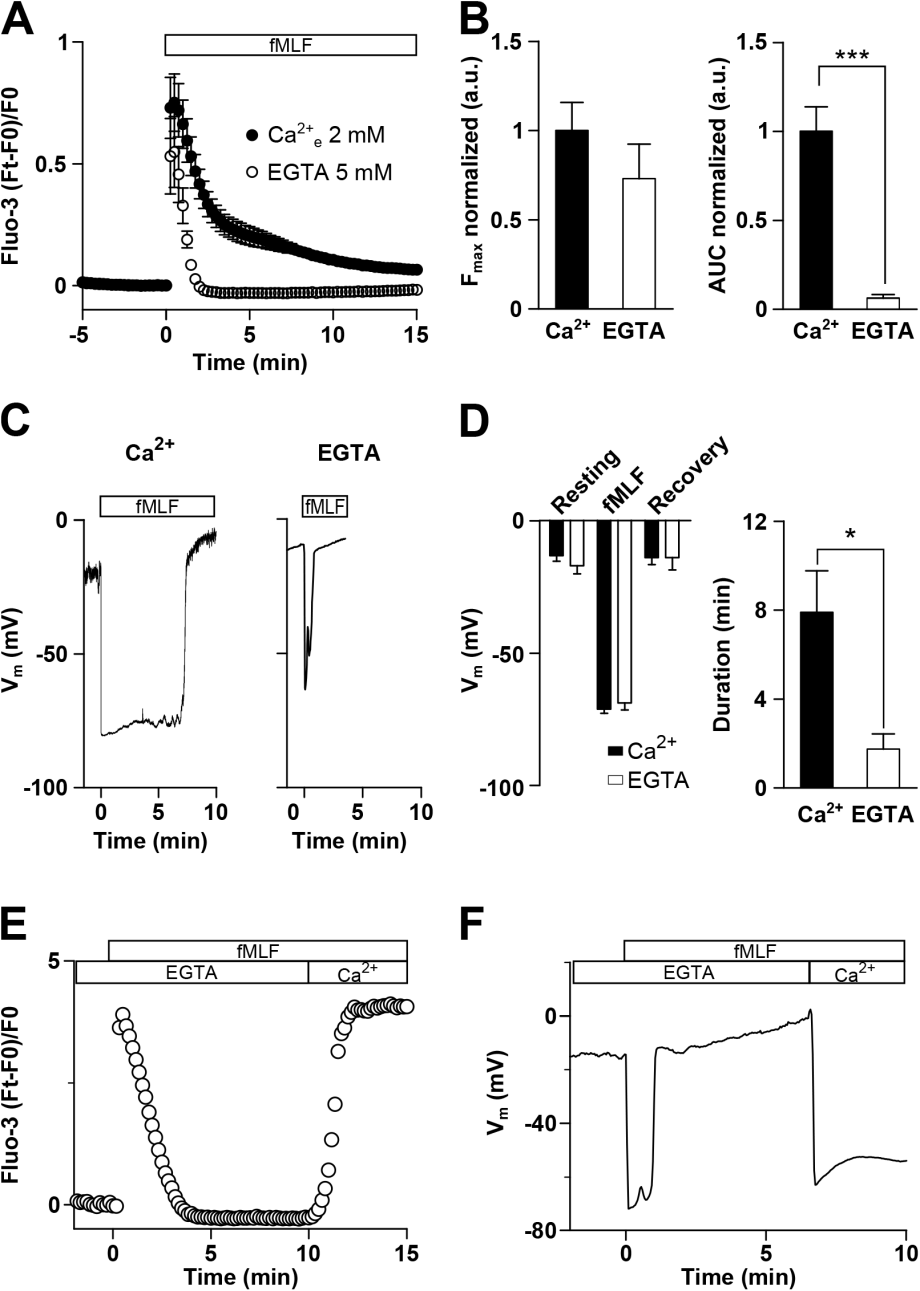
fMLF increases [Ca^2+^]_i_ and hyperpolarizes U937 cells. **A.** Average of Fluo-3 recordings in U937 cells in the presence of 2 mM Ca^2+^ (●) or absence of external Ca^2+^ plus 5 mM EGTA (○). fMLF (100 nM) was applied in the bath solution during the time indicated by the horizontal bar. **B.** Summarized data showing fMLF-induced maximum fluorescence increase in the presence or absence of external Ca^2+^ (Ca^2+^: 1.00 ± 0.16; 0 Ca^2+^: 0.73 ± 0.19, n = 8 and 6 for each condition, respectively, *p* > 0.05, left panel) and fMLF-induced AUC of the Ca^2+^ increase in the presence and absence of external Ca^2+^ (Ca^2+^: 1.00 ± 0.14; 0 Ca^2+^: 0.06 ± 0.02, n = 8 and 6 for each condition, respectively, *** *p* < 0.001, right panel). **C.** Representative traces of V_m_ recordings in U937 cells in a bath solution containing 2 mM Ca^2+^ (left panel) or in the absence of Ca^2+^ plus 5 mM EGTA (right panel). fMLF (100 nM) was applied during the time indicated by the horizontal bar. **D.** Summarized data showing fMLF-induced V_m_ changes in 2 mM of Ca^2+^ or in the absence of Ca^2+^ (Resting: -13.2 ± 2 mV (Ca^2+^) and -17.0 ± 3 mV (0 Ca^2+^); fMLF: -71.1 ± 2 mV (Ca^2+^) and -68.8 ± 3 mV (0 Ca^2+^); Recovery: -14.0 ± 3 mV (Ca^2+^) and -14.0 ± 5 mV (0 Ca^2+^), n = 3–22 for each condition, left panel) and the duration of fMLF-induced hyperpolarization in the presence or absence of external Ca^2+^ (Ca^2+^: 7.9 ± 2 min; 0 Ca^2+^: 1.8 ± 1 min, n = 15 and 4, respectively, * *p* < 0.05, right panel). **E** and **F.** Representative traces of Fluo-3 **(E)** and V_m_ **(F)** records in U937 cells stimulated with 100 nM fMLF as indicated by the horizontal bars. The cells were initially bathed without Ca^2+^ plus 5 mM EGTA and afterward bathed with a solution containing 2 mM Ca^2+^ as indicated by horizontal bar.

To monitor changes in [Ca^2+^]_i_ induced by fMLF, differentiated U937 cells were loaded with Fluo-3. As depicted in Fig 1A, fMLF (100 nM) in the presence of 2 mM external Ca^2+^ induced a rapid increase in intracellular Ca^2+^ followed by a slower decay phase. Under 0 external Ca^2+^ conditions (no Ca^2+^ added + 5 mM EGTA) the slower decay phase disappears. As shown in Fig 1B left panel, normalized peak fMLF-induced increase in [Ca^2+^]_i_ in the absence of external Ca^2+^ was not different to that observed with a solution containing 2 mM Ca^2+^. However, the normalized area under the curve (AUC) for Ca^2+^ increase was 94% reduced in the absence of external Ca^2+^ (Fig 1B right panel).

As previously reported, fMLF hyperpolarizes the transmembrane potential (V_m_) in macrophages [10]. Thus, we asked whether fMLF induces changes in V_m_ in differentiated U937 cells. Fig 1C depicts representative current clamp records in the presence of 2 mM external Ca^2+^ (left panel) or 0 external Ca^2+^ conditions (right panel). As shown, cells responded with a rapid (Ca^2+^: -0.131 ± 0.019 mV/ms; 0 Ca^2+^: -0.079 ± 0.005 mV/ms) hyperpolarization upon exposure to 100 nM fMLF reaching ~ -70 mV. Despite continuous exposure to fMLF V_m_ repolarizes rapidly after approximately 8 min. However, in the absence of external Ca^2+^ the duration of the hyperpolarization was 4-fold shorter (left panel) without a significant difference in the peak V_m_ reached. Fig 1D shows an average of the data from experiments similar to those shown in Fig 1C. Of note, in non-differentiated U937 cells (i.e. not treated with dibutyryl cAMP), fMLF was unable to induce changes in intracellular Ca^2+^ and V_m_ (S1 Fig), suggesting that the observed effect is dependent upon expression of the FPR as it has been shown that non-differentiated U937 cells do not express them [24].

To determine whether the [Ca^2+^]_i_ increase and hyperpolarization induced by fMLF followed a similar time course, differentiated U937 cells were exposed to fMLF (100 nM) in the absence of external Ca^2+^ for >5 min, then bathed with a solution containing 2 mM Ca^2+^. As depicted in Fig 1E and 1F, transient increase in [Ca^2+^]_i_ and hyperpolarization followed a similar temporal course, suggesting that both cellular responses might be linked by a common molecular mechanism.

### fMLF-dependent hyperpolarization is secondary to a Ca^2+^-dependent K^+^ conductance

**Fig 2:**
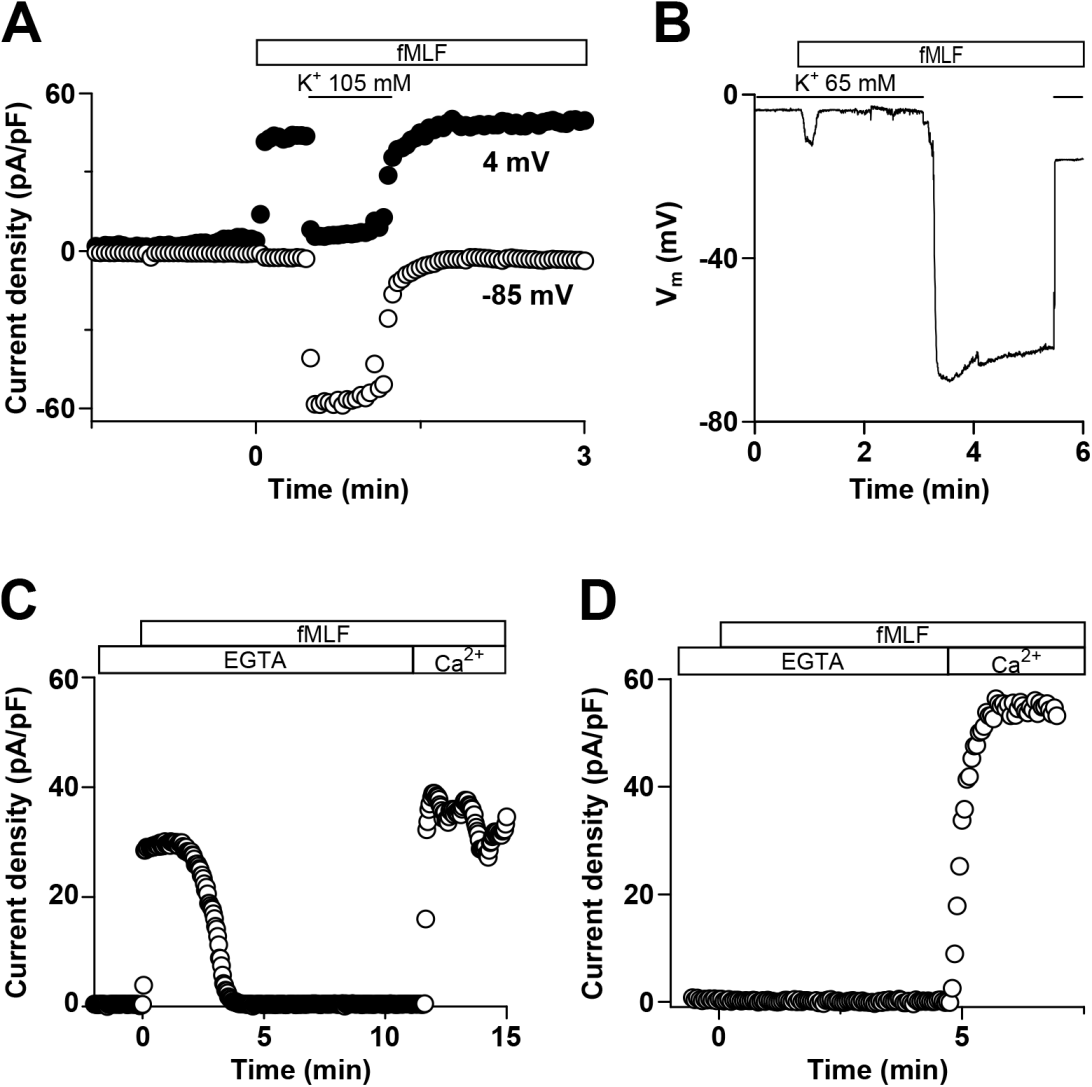
fMLF-dependent hyperpolarization is secondary to a Ca^2+^-dependent K^+^ conductance. **A.** Representative trace of a voltage-clamp recording showing the current density at -85 mV (○) and 4 mV (●) in U937 cells. Currents were measured using a holding potential of -30 mV and pulsing to -85 mV or 4 mV in square pulses of 0.5 s. The period for this pulse protocol was 2.5 s. Horizontal bar and black line indicate perfusion with 100 nM fMLF and a bath solution containing 105 mM K^+^, respectively. **B.** Representative trace of a current-clamp record showing V_m_ changes in U937 cells. Horizontal bar and black lines indicate perfusion with 100 nM fMLF and a bath solution containing 65 mM K^+^, respectively. **C** and **D**. Voltage-clamp recordings showing the currents at 4 mV in non-treated **(C)** and treated **(D)** U937 cells with 1 μM thapsigargin 5 min before exposure to fMLF. Same as **1E** and **1F**; cells were sequentially bathed without Ca^2+^ plus 5 mM EGTA and a solution containing 2 mM Ca^2+^ as indicated by horizontal bars.

Because fMLF treatment changed V_m_ to a value compatible with the reversal potential for K^+^ (E_K_+), we studied whether a K^+^ conductance underlies the fMLF-induced hyperpolarization. Fig 2A shows a representative voltage-clamp record of an experiment performed to determine the ionic nature of the hyperpolarizing current. To that end, differentiated U937 cells were stimulated with 100 nM fMLF and the current was recorded at E_K_+ (-85 mV) and E_Cl_- (4 mV), alternately. Therefore, if fMLF activates a K^+^ current, this current has to be observed at 4 mV. We observed an outwardly directed current of 33.20 ± 4.59 pA/pF at 4 mV. Furthermore, this current was completely abolished when cells were exposed to a bath solution containing 105 mM K^+^, which modified E_K_+ to a value close to 4 mV. Next, to confirm whether a K^+^ conductance underlies this hyperpolarization, we performed current clamp experiments, as shown by a representative current clamp record in Fig 2B. Cells where first exposed to 100 nM fMLF in a high-K^+^ (65 mM) external solution and then to a normal-K^+^ (5 mM) external solution. As shown, fMLF produced a small and brief hyperpolarization in high-K^+^ conditions, whereas under normal-K^+^ conditions a robust hyperpolarization was recorded. Furthermore, experiments performed with external tetraethylammonium (TEA 20 mM) and 140 mM internal Cs^+^ in order to block K^+^ conductances, yielded the same result as observed under external high-K^+^ conditions (S2 Fig).

Because the fMLF-induced hyperpolarization was found to be dependent on [Ca^2+^]_i_ as well as the similarity of the time course of Ca^2+^ and V_m_ changes, we hypothesized that the K^+^ conductance activated upon fMLF exposure should be also Ca^2+^ dependent. Fig 2C and 2D depict representative voltage-clamp recordings designed to study the Ca^2+^ dependence of the K^+^ current. In the absence of external Ca^2+^, the K^+^ current was briefly activated by fMLF (100 nM) and was reactivated upon reintroduction of 2 mM Ca^2+^ (Fig 2C). In addition, after depletion of intracellular Ca^2+^ reservoirs with thapsigargin (1 μM), activation of the K^+^ current upon fMLF exposure was not detected in the absence of external Ca^2+^. However, upon external Ca^2+^ reintroduction, the K^+^ current was rapidly activated (Fig 2D).

### K_Ca_3.1 is the K^+^ conductance activated by fMLF in U937 cells

**Fig 3:**
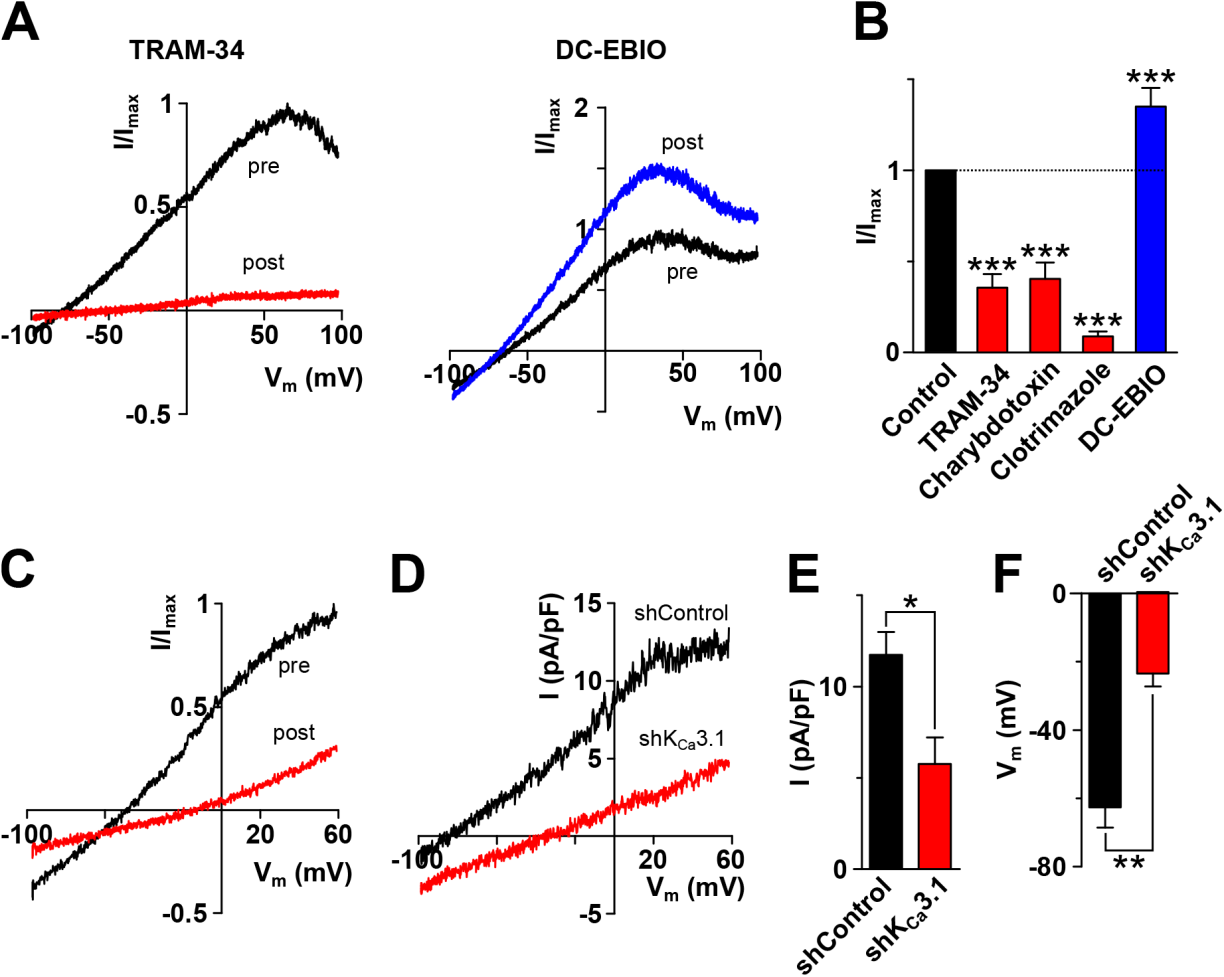
K_Ca_3.1 is the K^+^ conductance activated by fMLF in U937 cells. **A.** Current traces recorded with a voltage-clamp ramp protocol (-100 to 100 mV) in U937 cells previous (pre) and after exposure (post) to 100 nM TRAM-34 (left panel) and 10 μM DC-EBIO (right panel). **B.** Summarized pharmacological data showing the effects of blockers and DC-EBIO after fMLF-induced current has reached a stable magnitude. (TRAM-34, 100 nM: 36 ± 7%; charybdotoxin, 100 nM: 40 ± 9%; clotrimazole, 10 μM: 9 ± 3%; and DC-EBIO, 10 μM: 135 ± 10% of the control current; n = 3–5 for each condition, one-way ANOVA, Bonferroni's *post-hoc* test, *** *p* < 0.001). **C.** Representative currents traces of the Ca^2+^-activated current (1 μM Ca^2+^ in the pipette) elicited by a ramp protocol (-100 to 60 mV) under control conditions (pre) and after exposure to 10 μM clotrimazole (post). **D.** Representative currents traces of the Ca^2+^-activated current (1 μM Ca^2+^ in the pipette) elicited by a ramp protocol (-100 to 60 mV) in sh-control (shControl) and sh-K_Ca_3.1 U937 cells (shK_Ca_3.1). **E.** Summarized data showing the effect of K_Ca_3.1 knock down in Ca^2+^-induced K^+^ currents (shControl: 11.7 ± 1.3 pA/pF; shK_Ca_3.1: 5.8 ± 1.5 pA/pF, n = 3–4 for each condition, respectively, * *p* < 0.05). **F.** Summarized data of the effect of K_Ca_3.1 knock down on fMLF-induced (100 nM) changes in membrane potential measured using the nystatin perforated patch-clamp technique (shControl: -62.3 ± 6 mV; shK_Ca_3.1: -23.5 ± 3.8 pA/pF, n = 4 for each condition, ** *p* < 0.01).

Immune cells express the intermediate and small-conductance Ca^2+^-dependent potassium channels (K_Ca_) [19, 20, 25–27]. To determine which K_Ca_ is activated by fMLF in U937 cells, we used a pharmacological approach. The intermediate-conductance K_Ca_ channel (K_Ca_3.1) is blocked specifically and with high affinity (nanomolar range) by TRAM-34 and non-specifically by charybdotoxin and clotrimazole and is potentiated by DC-EBIO [28]. On the other hand, the small-conductance K_Ca_ channels (K_Ca_2.1, K_Ca_2.2 and K_Ca_2.3) are insensible to TRAM-34, charybdotoxin and clotrimazole, although enhanced by DC-EBIO [28]. Fig 3A show current traces recorded with a voltage-clamp ramp protocol (-100 to 100 mV) in U937 cells. As depicted (left panel), the current elicited by fMLF (E_rev_ -78.5 mV; E_K_+ -85 mV) was blocked (64%) by 100 nM TRAM-34 and potentiated (35%) by 10 μM DC-EBIO (right panel), suggesting that the molecular entity underlying fMLF-induced K^+^ conductance in these cells is K_Ca_3.1. Fig 3B summarizes the effect of K_Ca_ blockers and DC-EBIO on the K^+^ current elicited by fMLF in U937 cells. We next tested whether intracellular Ca^2+^ could directly activate currents in U937 cells. To that end, conventional whole cell experiments were performed with 1 μM Ca^2+^ in the pipette solution. Fig 3C depicts representative current traces elicited by a ramp protocol (-100 to 60 mV) in the presence and absence of 10 μM clotrimazole. As shown, clotrimazole inhibited the Ca^2+^-activated current, confirming the presence of a Ca^2+^-activated K^+^ current in these cells. For unambiguous identification of the molecular identity of this Ca^2+^-activated K^+^ current, we performed knock down experiments. Using the same experimental conditions as described above, lentiviral transduced cells with either a control sh (scrambled sequence) or a set of three K_Ca_3.1-targeted sh were used. As depicted in Fig 3D and 3E, the Ca^2+^-activated current K^+^ induced by 1 μM intracellular Ca^2+^ was decreased in cells carrying the shK_Ca_3.1. Furthermore, as shown in Fig 3F fMLF-induced hyperpolarization was blunted in shK_Ca_3.1 cells.

### K_Ca_3.1 operates as a positive feedback mechanism for intracellular Ca^2+^ increase

**Fig 4:**
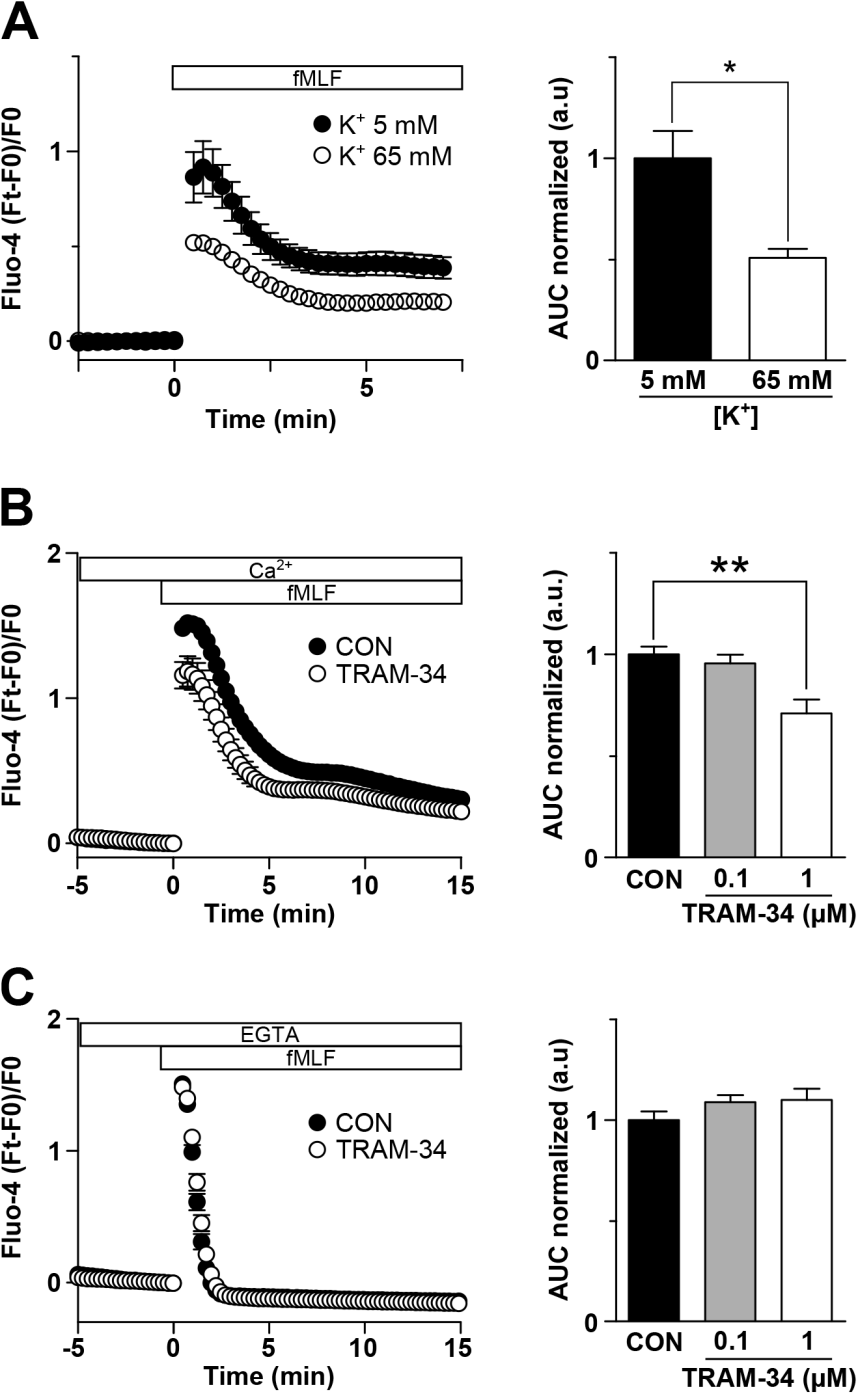
K_Ca_3.1 operates as a positive feedback mechanism for intracellular Ca^2+^ increase. Average of Fluo-4 records during fMLF treatment (left panels) and AUC of fMLF-induced Ca^2+^ increase (right panels) in U937 cells in three different conditions. **A.** Cells were treated with fMLF in the presence of 5 mM (●) or 65 mM (○) external K^+^. AUC was 1 ± 0.14 with 5 mM K^+^ and 0.51 ± 0.04 with 65 mM K^+^ (n = 3, * *p* < 0.05). **B** and **C.** Cells were exposed to 1 μM TRAM-34 (○) or control condition (CON, ●). AUC was calculated both in CON and two different concentrations of TRAM-34 (0.1 and 1 μM). In **(B),** AUC was determined in the presence of 2 mM Ca^2+^ (CON: 1 ± 0.04; TRAM-34, 0.1 μM: 0.96 ± 0.04; TRAM-34, 1 μM: 0.71 ± 0.07, n = 6, one-way ANOVA Bonferroni's *post hoc* test, ** *p* < 0.01). In **(C)**, AUC was measured in the absence of Ca^2+^ plus 5 mM EGTA (CON: 1 ± 0.04; TRAM-34, 0.1 μM: 1.1 ± 0.03; TRAM-34, 1 μM: 1.1 ± 0.05, n = 6).

Finally, we explored whether the hyperpolarization mediated by K_Ca_3.1 enhances the increase in [Ca^2+^]_i_, by modifying the driving force for Ca^2+^ influx. Fig 4 displays intracellular Ca^2+^ monitoring experiments showing the effect of TRAM-34 and external high-K^+^ on fMLF-induced Ca^2+^_i_ dynamics. Fig 4A depicts the Ca^2+^_i_ time course (left panel) and the normalized AUC (right panel) in normal K^+^ (5 mM) and high-K^+^ (65 mM, calculated E_K_+ -20 mV) external solutions. As shown, fMLF-induced Ca^2+^_i_ increase is reduced (~49%) in high-K^+^. On the other hand, 1 μM TRAM-34 inhibited (~29%) fMLF-induced Ca^2+^_i_ increase (Fig 4B, left and right panel). Furthermore, TRAM-34 prevented fMLF-induced hyperpolarization (S3 Fig). In the absence of external Ca^2+^ the increase in [Ca^2+^]_i_ was not affected by TRAM-34 (Fig 4C, left and right panel), indicating that K_Ca_3.1-dependent hyperpolarization induced by fMLF enhances the increase of [Ca^2+^]_i_ by an augmented Ca^2+^ influx.

## Discussion

In this study, we demonstrate that fMLF induces in differentiated human U937 cells a Ca^2+^-dependent hyperpolarization via activation of the K^+^ channel K_Ca_3.1. In addition, we found that this hyperpolarization enhances fMLF-induced intracellular Ca^2+^ increase through an amplification of the driving force for Ca^2+^ entry. Consequently, enhanced Ca^2+^ influx would in turn lengthen hyperpolarization, operating as a positive feedback mechanism for fMLF-induced signaling.

Our data demonstrate that plasma membrane hyperpolarization induced by fMLF in U937 cells is due to the activation of a K^+^ current as V_m_ reaches -71 mV, a value close to the E_K_^+^ (-85 mV). In addition, when [K^+^]_e_ is increased to 65 mM, which changes E_K_^+^ to -20 mV, the magnitude of the hyperpolarization is significantly decreased. We also found that this K^+^ conductance corresponds to a Ca^2+^-dependent K^+^ channel because the activation of the current follows the same temporal course of the intracellular Ca^2+^ increase. Also, absence of Ca^2+^ influx and inhibition of Ca^2+^ release from intracellular reservoirs hindered the activation of the fMLF-induced K^+^ conductance. Finally, based on the pharmacological profile (inhibition by TRAM-34 and potentiation by DC-EBIO) [28, 29] as well as functional knock down we identified, for the first time, K_Ca_3.1 as the molecular entity responsible for fMLF-induced K^+^ current in macrophage-like differentiated U937 cells.

fMLF-induced Ca^2+^ increase occurs via release from intracellular reservoirs and Ca^2+^ influx. Several studies report that upon fMLF binding to its receptor Ca^2+^ is released from intracellular reservoirs trough IP_3_ receptor activation [4, 7, 12]. This release promotes an initial, fast and transient increase in [Ca^2+^]_i_ followed by a sustained phase dependent on Ca^2+^ influx. Although we did not explore the molecular entities underlying fMLF-induced Ca^2+^ entry, it is interesting to note that clotrimazole is almost 3-fold more effective in reducing fMLF-induced Ca^2+^ increase than TRAM-34, a value similar to that observed under 0 external Ca^2+^ plus EGTA. The robust effect of clotrimazole on the Ca^2+^ increase (S4 Fig) could be explained by the fact that clotrimazole besides blocking K^+^ conductances also blocks TRPM2, known to be expressed in U937 cells [30], in the same dose range [28, 31]. In addition, store operated channel entry (SOCE) might play a role in the influx of Ca^2+^, as K_Ca_3.1 and SOCE are known to be tightly coupled in human macrophages [32].

Immune cell function depends on the magnitude and duration of the increase in the [Ca^2+^]_i_ [12, 33]. The driving force for Ca^2+^ entry is a critical factor to boost Ca^2+^ signaling. In our experiments, fMLF shifts V_m_ from -13 mV to -71 mV, implying roughly a 44% increase in the driving force for Ca^2+^ entry. Because K_Ca_3.1 is responsible for the hyperpolarization and, consequently, the increase in the driving force, the inhibition of this channel significantly decreases intracellular Ca^2+^ increase. This decrease occurs only in the presence of external Ca^2+^, indicating that Ca^2+^ entry is decreased by a reduction in the driving force. In fact, we observed that in the presence of external Ca^2+^ K_Ca_3.1 inhibition by 1 μM TRAM-34 decreased fMLF-induced Ca^2+^ increase by 29% (Fig 4B). Interestingly, a similar result was observed in microglial cells, in which K_Ca_3.1 inhibition by TRAM-34 (1 μM) was found to reduce the increase of Ca^2+^_i_ triggered by extracellular UTP in approximately 30% [22].

As mentioned above K_Ca_3.1 has a role in the Ca^2+^ signaling induced by fMLF. Thus, the maximal effect of the K_Ca_3.1 blocker on Ca^2+^ signaling should be observed at a concentration sufficient to abolish fMLF-dependent hyperpolarization. However, we observed that to block the fMLF-induced hyperpolarization 0.1 μM TRAM-34 was enough, while the increase in the [Ca^2+^]_i_ triggered by fMLF was only significantly diminished with 1 μM TRAM-34. Despite the inconsistency regarding the concentration required to block fMLF-induced hyperpolarization and Ca^2+^ increase, our results do support a relevant role of K_Ca_3.1. It should be noted that fMLF-induced Ca^2+^ signaling was diminished by 49% when hyperpolarization was prevented by increasing [K^+^]_e_, which confirms that half of the increase in the [Ca^2+^]_i_ is mediated by a K^+^-dependent hyperpolarization. Also, although TRAM-34 at 1 μM is still considered a specific blocker of K_Ca_3.1, at this concentration TRAM-34 could block other voltage-gated K^+^ channels, which have an IC_50_ of approximately 5 μM [29]. Therefore, it can be concluded that the effect of TRAM-34 on Ca^2+^ signaling is mediated by K_Ca_3.1, although we can not discard the role of other K^+^ channels.

It has been previously shown that K_Ca_3.1 plays several roles in the immune response [34–36]. For example, K_Ca_3.1 facilitates mast cell degranulation in mice [35], migration of dendritic cells [34] and promotes atherogenesis in mice due to an infiltration by macrophages and T lymphocytes in plaques [36]. All of the above mentioned processes share a common mechanism; an enhancement in Ca^2+^_i_ increase by boosting the driving force for Ca^2+^ entry. Therefore, our study suggests that K_Ca_3.1 might enhance chemotaxis, phagocytosis, reactive oxygen species as well as cytokine production in monocytes and macrophages upon stimulation with chemotactic peptides.

In summary, our data indicate that K_Ca_3.1 is the molecular entity responsible for the hyperpolarization observed upon fMLF exposure in differentiated U937 cells, thereby controlling the driving force for Ca^2+^ entry and thus, modulating Ca^2+^_i_ signaling in these cells through a positive feedback mechanism.

## Supporting Information

S1 Fig Non-differentiated U937 cells do not respond to fMLF stimulation. **A** and **B.** Representative Indo-1 (**A**) and V_m_ (**B**) recordings during fMLF treatment in non-differentiated U937 cells, 100 nM fMLF and 5 μM ionomycin (ION) were applied during the time indicated by the horizontal bars.
(EPS)

S2 Fig Extracellular TEA plus intracellular Cs^+^ prevent fMLF-induced hyperpolarization in U937 cell. V_m_ was recorded in a differentiated U937 cell in the presence of 20 mM TEA in the bath and 140 mM Cs^+^ in the pipette. Horizontal bar indicates exposure to 100 nM fMLF (n = 4).
(EPS)

S3 Fig TRAM-34 prevents fMLF-induced hyperpolarization in U937 cells. V_m_ recording obtained form a differentiated U937 cell in the presence of 100 nM TRAM-34 in the bath. Horizontal bar indicates exposure to 100 nM fMLF (n = 4).
(EPS)

S4 Fig Clotrimazole decreases fMLF-induced Ca^2+^i increase in U937 cells. **A.** Average of Indo-1 records performed in control conditions (●) or in the presence of 10 μM clotrimazole (○) in differentiated U937 cells. In both conditions cells were treated with 100 nM fMLF during the time indicated by the horizontal bar. **B.** AUC of fMLF-induced Ca^2+^ increase in control (black) or clotrimazole (empty) treatment (Control: 1 ± 0.16; clotrimazole, 10 μM: 0.22 ± 0.08, n = 6 and 3, respectively, * *p* < 0.05).
(EPS)
